# Machine Learning Assisted Prediction of Prognostic Biomarkers Associated With COVID-19, Using Clinical and Proteomics Data

**DOI:** 10.3389/fgene.2021.636441

**Published:** 2021-05-20

**Authors:** Rahila Sardar, Arun Sharma, Dinesh Gupta

**Affiliations:** ^1^Translational Bioinformatics Group, International Centre for Genetic Engineering and Biotechnology, New Delhi, India; ^2^Department of Biochemistry, Jamia Hamdard, New Delhi, India

**Keywords:** machine learning, biomarkers discovery, COVID-19, feature selection, proteomics and bioinformatics

## Abstract

With the availability of COVID-19-related clinical data, healthcare researchers can now explore the potential of computational technologies such as artificial intelligence (AI) and machine learning (ML) to discover biomarkers for accurate detection, early diagnosis, and prognosis for the management of COVID-19. However, the identification of biomarkers associated with survival and deaths remains a major challenge for early prognosis. In the present study, we have evaluated and developed AI-based prediction algorithms for predicting a COVID-19 patient’s survival or death based on a publicly available dataset consisting of clinical parameters and protein profile data of hospital-admitted COVID-19 patients. The best classification model based on clinical parameters achieved a maximum accuracy of 89.47% for predicting survival or death of COVID-19 patients, with a sensitivity and specificity of 85.71 and 92.45%, respectively. The classification model based on normalized protein expression values of 45 proteins achieved a maximum accuracy of 89.01% for predicting the survival or death, with a sensitivity and specificity of 92.68 and 86%, respectively. Interestingly, we identified 9 clinical and 45 protein-based putative biomarkers associated with the survival/death of COVID-19 patients. Based on our findings, few clinical features and proteins correlate significantly with the literature and reaffirm their role in the COVID-19 disease progression at the molecular level. The machine learning–based models developed in the present study have the potential to predict the survival chances of COVID-19 positive patients in the early stages of the disease or at the time of hospitalization. However, this has to be verified on a larger cohort of patients before it can be put to actual clinical practice. We have also developed a webserver CovidPrognosis, where clinical information can be uploaded to predict the survival chances of a COVID-19 patient. The webserver is available at http://14.139.62.220/covidprognosis/.

## Introduction

In December 2019, the COVID-19 disease initiated as an outbreak caused by SARS-CoV-2, which quickly snowballed into a catastrophic worldwide healthcare crisis (Srivastava et al., 2020). On March 11, 2020, the World Health Organization (WHO) declared COVID-19 a global pandemic with more than 118,000 cases in 114 countries and over 4,000 deaths, much more than the morbidity and mortality caused by related viruses such as SARS and MERS. As of March 14, 2021, the pandemic has caused more than 119 million confirmed COVID-19 cases and ∼2.64 million deaths worldwide^1^.

Compared to other respiratory diseases such as influenza, the COVID-19 human-to-human transmission is facilitated through respiratory droplets (particles > 5–10 nm in diameter) from coughing and sneezing. The clinical symptoms associated with COVID-19 patients vary from asymptomatic or symptomatic forms (Cascella et al., 2020). A study published in *JAMA* consists of data from 72,314 cases, including records from confirmed, suspected, diagnosed, and asymptomatic COVID-19 patients, shared by the Chinese Center for Disease Control and Prevention (China CDC), demonstrating the epidemiologic curve of the Chinese outbreak. As per this report, the mortality of critically ill patients was 49.0% in contrast to 2.3% for the overall COVID-19 patients. The mortality was also higher for patients with various comorbidities such as cardiovascular disease, diabetes, chronic respiratory disease, and oncological diseases, whereas patients with the age of 9 or younger did not have any fatal cases (Wu and McGoogan, 2020).

At present, no SARS-CoV-2 specific drug or reliable prognostic biomarker is available for COVID-19 treatment (González-Pacheco et al., 2020; Pandey et al., 2020). Various therapeutic measures to enhance the immune systems by immune modulators have been proposed (Zhong et al., 2020). Recommended preventive measures include social distancing, proper health, and hygiene management (Al-Rohaimi and Al Otaibi, 2020). It is also known that the severity of COVID-19 largely depends on the host and viral factors. The latter highlights the importance of identifying the host features associated with the disease severity at the molecular level (Zhang et al., 2020). Given the facts enumerated above, it is desirable to have the correct prognostic assessment of patients for proper clinical management.

Artificial intelligence (AI) is being employed to meet new healthcare requirements, in view of the pandemic, for example, tracking the SARS-CoV-2 virus spread and quickly identifying high-risk patients (Sharma et al., 2020). Machine learning (ML) methods have been exploited to analyze various kinds of biological datasets such as proteomics data, NGS data, and metabolomics data to predict the biomarkers for classification of samples and genes associated with a particular disease state (Dumancas et al., 2017; Cambiaghi et al., 2018). The mitigation potential of AI technology has been extensively demonstrated for various pandemics and infectious diseases, for example, SARS, Ebola, HIV, and COVID-19 (Lalmuanawma et al., 2020; Overmyer et al., 2020).

To date, there are several reports on clinical biomarkers associated with the disease prognosis. However, there are only a few published articles on protein-based biomarkers, and hence, further research is required to confirm the existing findings (Graziani et al., 2020; Kaur et al., 2020; Kermali et al., 2020). Integrated data analysis on COVID-19 genomes has been performed to identify several crucial factors involved in host–pathogen interaction. However, limited attempts have been made to integrate high throughput datasets (Sardar et al., 2020). Yan et al. (2020b) developed a machine learning model with more than 90% accuracy on 485 COVID positive patients to predict the clinical biomarkers associated with individual patients’ mortality. Another study by Yao H. et al. (2020) aimed to predict the disease severity among the patients by utilizing the data on 137 COVID-19 infected patients using an ML-based model on the blood and urine examination parameters. However, these methods are not free from errors, limitations, and challenges, rendering them unfit to be used in real-world problems.

Motivated by the availability of appropriate clinical datasets, we used such a dataset for training ML algorithms to exploit its potential for the prognosis of COVID-19 positive patients. We designed a pipeline to predict features, namely proteins and clinical parameters, associated with the disease severity and survival of the COVID-19 patients. Interestingly, we have identified 9 clinical features and 45 proteins related to the survival/death of COVID-19 patients. Few of the identified clinical features and proteins correlate well with the literature and reaffirm their role in the COVID-19 disease progression at the molecular level (Shen et al., 2020; Wynants et al., 2020; Yan et al., 2020a). The potential role of identified proteins in various pathways, their native functions, potential to be a drug target, etc., are described in the subsequent sections. The ML-based models developed in the present study possess an immense potential to predict the survival chances of COVID-19 positive patients in the early stages of the disease or at the time of hospitalization.

## Materials and Methods

### Data Source

We downloaded the clinical and normalized protein expression profile data for 306 COVID-19 patients and 78 other patients (control subjects) from the Olink website (Filbin et al.). We downloaded three files, namely “MGH_COVID_OLINK_NPX.txt,” “MGH_COVID_Clinical_ Info.txt,” and “variable_descriptions.xlsx,” containing protein data (with relative quantification values given in Olink’s proprietary Normalized Protein expression (NPX) units), essential clinical data (associated with each sample), and a worksheet (with a description of the clinical variables presented), respectively. Although clinical and protein data were present in two different files, the data were linked based on the subject IDs.

### Data Preprocessing

**FIGURE 1:**
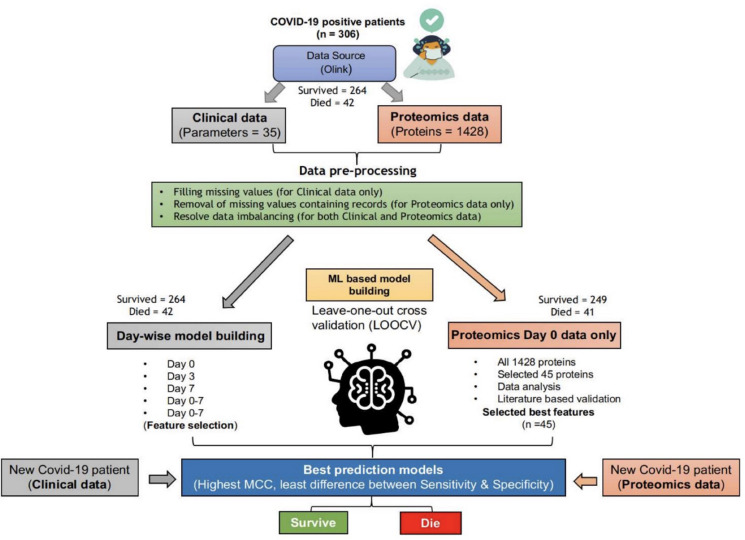
ML-based pipeline to identify key features associated with survival based on clinical and proteomics data. (The figure images were generated using biorender.com).

Data preprocessing is essential for a machine learning study. Hence, we checked the data for any experimental impurities through semiautomated ways. As depicted in Figure 1, clinical and proteomic data were missing for a few patients. In the case of clinical data, we replaced missing values with “-1.” Thus, we used the clinical data of 42 dead and 264 survivors (Whole dataset I) for training the “Clinical Information” based classification models for days 0–7. However, in the proteomics data, the protein expression values were missing for 165 and 248 patients for days 3 and 7, respectively. Therefore, we used only proteomics data for the Day 0 proteomics information-based classification model generation. For only one COVID-19 positive patient (who died within 28 days of hospitalization), protein expression values (for few of the 1,428 proteins) were missing, while protein expression values were missing for 15 patients among the survivors (for few of the 1,428 proteins); hence, we excluded these records from the study (Figure 1). Thus, we used the proteomics data (Whole dataset II) of 41 dead and 249 survivors to train and validate the machine learning–based models.

As evident from the downloaded data, the number of survivors and deaths in clinical as well as proteomics data were imbalanced. The survivor’s data (for both clinical and proteomics data) were split into five, almost equal-sized, divisions (P1–P5). Furthermore, we trained and validated the models using each of the five divisions and the dataset of dead patients. The tools, techniques, and statistical measures used to evaluate the model performances and the retrieved results are given in the subsequent sections.

### Tools Used for the Development of Classification Models

WEKA (Frank et al., 2016), a popular and widely used data mining and machine learning tool, was used for training and validation of the various machine learning–based classification models developed in this study. All the techniques available with the WEKA (v3.8.2) were used to train and validate the classification models. For clinical data, five types of models are generated, i.e., the models based on (1) Day 0 clinical parameters, (2) Day 3 clinical parameters, (3) Day 7 clinical parameters, (4) Days 0–7 clinical parameters, and (5) Selected clinical parameters (out of Days 0–7 clinical parameters). On the other hand, for proteomic data, two types of models are generated, i.e., (1) Day 0, all 1428 protein parameters, and (2) Day 0 protein parameters based on feature selection.

We trained and evaluated 44 different types of ML classification algorithms available in WEKA (v3.8.2). However, several combinations of various parameters for these algorithms and the number of input parameters used (for the training and validation of classification models) resulted in thousands of models (for details, check http://14.139.62.220/covidprognosis/supple.php). For example, in the case of Day 0 clinical parameters-based model (using the P1 dataset), a total of 85 models were trained and evaluated using Day 0 all 33 clinical parameters. Thus, for P1–P5 splits, a total of 425 models (85 × 5) were developed to determine the best classification models.

### Feature Selection

In different machine learning–based classification studies, all the input features do not play an equally significant role in classification (Sharma et al., 2016; Jablonka et al., 2020; Kumar et al., 2020). Therefore, to identify the most significant clinical and proteomics features, all the feature selection techniques available with WEKA were applied to the Days 0–3 clinical features dataset (consisting of 33 clinical parameters) and Day 0 proteomics data (for the 1,428 proteins).

### Cross-Validation Techniques Used

The availability of enormous data is essential for preparing training and validation datasets during a machine learning–based study. However, due to limited patients’ records, it was impossible to prepare separate training and validation datasets. Therefore, the leave-one-out cross-validation (LOOCV) technique was used to utilize the available information optimally. In the LOOCV technique, the models are trained and validated so that each record is used for training and testing. The LOOCV technique has widely been used to solve several classification problems (Mete et al., 2016; Nath and Subbiah, 2016; Jiang et al., 2019).

### Formulae Used to Evaluate Performance of the Models

The performance of the models was evaluated using statistical measures such as sensitivity, specificity, accuracy, and Mathew’s correlation coefficient (MCC). The formulae used are given below:

$$\mathrm{Sensitivity}=\frac{\mathrm{TP}}{\mathrm{TP}+\mathrm{FN}}\times 100$$

$$\mathrm{Specificity}=\frac{\mathrm{TN}}{\mathrm{TN}+\mathrm{FP}}\times 100$$

$$\mathrm{Accuracy}=\frac{\mathrm{TP}+\mathrm{TN}}{\mathrm{TP}+\mathrm{FP}+\mathrm{TN}+\mathrm{FN}}\times 100$$

$$\mathrm{MCC}=\frac{\left(\mathrm{TP}\right)\left(\mathrm{TN}\right)-\left(\mathrm{FP}\right)\left(\mathrm{FN}\right)}{\sqrt{\left[\mathrm{TP}+\mathrm{FP}\right]\left[\mathrm{TP}+\mathrm{FN}\right]\left[\mathrm{TN}+\mathrm{FP}\right]\left[\mathrm{TN}+\mathrm{FN}\right]}}\times 100$$

where TP and TN are correctly predicted positive and negative examples, respectively. Similarly, FP and FN are wrongly predicted positive and negative examples, respectively. The models with the highest MCC value and almost equal sensitivity and specificity values are considered best prediction models.

### Pathway Analysis and Identification of Drug Targets

To understand the biological functions of the shortlisted proteins, pathway analysis was performed using the DAVID tool (Jiao et al., 2012). Targeting host proteins appears to be a promising approach in antiviral research. To identify the drugs against the selected proteins, all the drug target information was downloaded from the TTD database, and only validated and clinically proven drugs were used for the analysis (Wang et al., 2020). The drugs that have been withdrawn or not in use were removed from the drug-targets based analysis.

### Webserver Development

The CovidPrognosis webserver has been developed using efficient and open-source Linux-Apache-MySQL-PHP/Perl/Python (LAMP) server technologies. The user interface (UI) or web interface is developed using HTML, CSS, PHP (v7.1.28), and AJAX. Moreover, the predictions are performed using the WEKA-based machine learning models, trained and validated on clinical parameters.

## Results

### Models Based on Whole Clinical Parameters

The classification models were developed using clinical information, as given in Supplementary Table 1. A total of five types of models (thousands in number; based on all available techniques in the WEKA package) were developed using the Day 0 (Sr. No. 3-21), Day 3 (Sr. No. 3-14 and 22-28), Day 7 (Sr. No. 3-14 and Sr. No. 29-35), and Days 0–7 (Sr. No. 3-35) clinical parameter values (Supplementary Table 1). However, two models achieved the highest performance using Day 0 and Days 0–7 information, while “Whole dataset I” based models showed a large difference between sensitivity and specificity values. This difference may be attributed to the imbalance between the number of records for survived and died patients. The Day 0 clinical parameters-based model (using the “IterativeClassifierOptimizer” technique) achieved a maximum accuracy of 87.37% with the highest sensitivity (%), specificity (%), MCC, and ROC values of 88.10, 86.79, 0.75, and 0.863, respectively (Table 1). Using “RandomForest” as the classification technique and Days 0–7 clinical parameters (33) as input features, a maximum accuracy of 89.47% was achieved with the highest sensitivity (%), specificity (%), MCC, and ROC values of 85.71, 92.45, 0.79, and 0.921, respectively (Table 1).

**TABLE 1:** Performance of best models based on whole clinical parameters.

Dataset (no. of clinical parameters used)	Day(s)	Sensitivity (%)	Specificity (%)	Accuracy (%)	MCC	ROC	WEKA technique used
Whole dataset I (19)	0	50	94.7	88.56	0.48	0.806	AttributeSelectedClassifier
**P1 (19)**	**0**	**88.1**	**86.79**	**87.37**	**0.75**	**0.863**	**IterativeClassifierOptimizer**
Average of P1–P5 splits (19)	0	81.90	82.94	82.48	0.65	0.808	IterativeClassifierOptimizer
Whole dataset I (33)	0, 3, 7	47.62	96.21	89.54	0.51	0.739	J48
**P2 (33)**	**0, 3, 7**	**85.71**	**92.45**	**89.47**	**0.79**	**0.921**	**RandomForest (with -K 4)**
Average of P1–P5 splits (33)	0, 3, 7	75.24	81.43	78.68	0.57	0.868	RandomForest (with -K 4)

### Feature Selection for Clinical Parameters

For the clinical data, three clinical parameters, namely, age, absolute lymphocyte count (Day 0), and creatinine level (Day 0), and nine clinical parameters, i.e., age, absolute lymphocyte count (Day 0), creatinine level (Day 0), preexisting heart disease(s), preexisting hypertension, preexisting kidney disease(s), D-dimer level (Day 0), any GI-related symptoms at the time of hospital presentation, and cardiac event-Trop_72 (hs-cTn = > 100 within the first 72 h of presentation) clinical parameters or features were selected by the majority of the techniques^2^. Therefore, these three clinical parameters (selected by CfsSubsetEval as “Attribute Evaluator” with BestFirst as “Search Method”) and nine clinical parameters [selected by “InfoGainAttributeEval” as “Attribute Evaluator” with Ranker algorithm (attributes with ranking value > 0 were selected)] have been used for the training and evaluation of the machine learning–based models.

### Models Based on Selected Clinical Parameters

**FIGURE 2:**
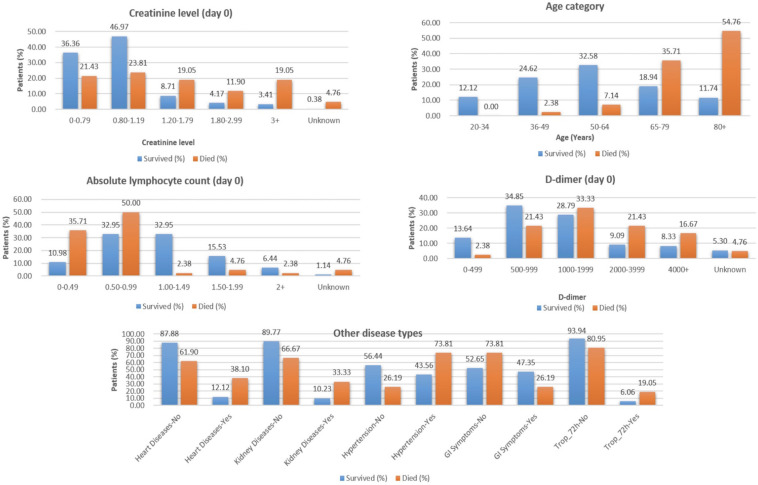
Selected features from clinical data to classify COVID-19 patients who survived vs. those who died.

From the analysis of the clinical data, it is found that the patients from the age group of 65–80+ years, with lower elevated lymphocyte count at Day 0 (<1.00), D-dimer ≥ 1,000 (units), are at a higher risk of death during hospitalization and require immediate treatment (Figure 2).

The “Whole dataset I”–based models showed a large difference between sensitivity and specificity values. A maximum accuracy of 87.37% was achieved with sensitivity (%), specificity (%), MCC, and ROC values of 85.71, 88.68, 0.74, and 0.845, from the three selected clinical features, respectively. While from the nine selected clinical parameters, a maximum accuracy of 86.32% was achieved with sensitivity (%), specificity (%), and MCC, and ROC values of 83.33, 88.68, 0.72, and 0.81, respectively, as shown in Table 2. The identified clinical features such as serum creatinine (Day 0), age, absolute lymphocyte count (Day 0), and D-dimer (Day 0) along with comorbidities such as preexisting heart disease(s), preexisting kidney disease(s), preexisting hypertension, GI symptoms at presentation, and Trop-72 can be highly useful in the classification of patients with survival or dying probabilities. These identified features can be evaluated as biomarkers that can help identify the patients who require immediate medical attention.

**TABLE 2:** Performance of best models based on selected clinical parameter values.

Dataset (no. of clinical parameters used)	Day(s)	Sensitivity (%)	Specificity (%)	Accuracy (%)	MCC	ROC	WEKA technique used
Whole dataset I (3)	0	50	94.7	88.56	0.48	0.806	J48
**P2 (3)**	**0**	**85.71**	**88.68**	**87.37**	**0.74**	**0.845**	**RandomSubSpace**
Average of P1–P5 splits (3)	0	83.33	80.31	81.64	0.63	0.831	RandomSubSpace
Whole dataset I (9)	0	50	94.7	88.56	0.48	0.806	AttributeSelectedClassifier
**P2 (9)**	**0, 3**	**83.33**	**88.68**	**86.32**	**0.72**	**0.81**	**IterativeClassifierOptimizer**
Average of P1–P5 splits (9)	0, 3	81.43	78.02	79.54	0.59	0.823	IterativeClassifierOptimizer

### Models Based on Whole NPX Proteomics Data

To understand the role of the protein expression profile in the classification of COVID-19 patients who survived vs. are dead, the expression values of 1428 proteins were used to develop machine learning–based classification models. The “Whole dataset II”–based models showed a large difference between sensitivity and specificity values. It is evident from Table 3 that an accuracy of 83.52% was achieved (using the dataset P4) with a sensitivity (%), specificity (%), MCC, and ROC values of 82.93, 84, 0.67, and 0.868, respectively.

**TABLE 3:** Performance of best models based on all 1428 proteins NPX values.

Dataset (no. of proteins used)	Day(s)	Sensitivity (%)	Specificity (%)	Accuracy (%)	MCC	ROC	WEKA technique used
Whole Dataset II (1428)	0	39.02	95.18	87.24	0.4	0.791	AdaBoostM1
P4 (1428)	0	82.93	84	83.52	0.67	0.868	LogitBoost
Average of P1–P5 splits (1428)	0	69.76	71.90	70.94	0.42	0.755	LogitBoost

### Identification of Proteins Associated With Survival vs. Deaths

The feature selection technique was applied to determine the most significant proteins that are helpful for the classification of patients who survived COVID-19 vs. those who died. Therefore, for proteomics data, different feature selection techniques resulted in the selection of a different set of proteomic features (see text footnote 2). Thus, a total of 45 proteins were identified through WEKA using CfsSubsetEval as the “Attribute Evaluator” with BestFirst as the “Search Method” (Supplementary Tables 2, 3).

As evident from Table 4, an accuracy of 89.01% was achieved (using the dataset P2) with sensitivity (%), specificity (%), MCC, and ROC values of 92.68, 86, 0.78, and 0.953, respectively. On the other hand, “Whole dataset II”–based models showed a large difference between sensitivity and specificity values.

**TABLE 4:** Performance of best models based on selected 45 protein NPX values.

Dataset (No. of proteins used)	Day(s)	Sensitivity (%)	Specificity (%)	Accuracy (%)	MCC	ROC	WEKA technique used
Whole dataset II (45)	0	80.49	92.77	91.03	0.67	0.948	BayesNet
**P2 (45)**	**0**	**92.68**	**86**	**89.01**	**0.78**	**0.953**	**BayesNet**
Average of P1–P5 splits (45)	0	82.44	82.72	82.59	0.65	0.902	BayesNet
**P5 (45)**	**0**	**85.37**	**91.84**	**88.89**	**0.78**	**0.886**	**SMO; NormalizedPolyKernel**
Average of P1–P5 splits (45)	0	83.42	79.97	81.51	0.63	0.817	SMO; NormalizedPolyKernel

### Expression and Pathway Analysis of the Shortlisted Proteins

The shortlisted proteins include lipid metabolism proteins (APOM), a protease inhibitor (FETUB), serine protease (FA7, GGH), growth factors (EGFR, PDGFB, TGFA, and GDF8), chemokines, interleukins (IL8, IL17C), and others (Supplementary Table 2). Recent studies have shown that APOM is downregulated in severe COVID-19 patients (Shen et al., 2020). The dysregulation of APOM is also associated with hepatitis B virus (HBV) infected patients (Gu et al., 2011). Another important protein associated with survival is angiopoietin (AGP), which is recently reported to cause inflammatory intussusceptive angiogenesis and diffuse alveolar damage in COVID-19, and the progression of carcinogenetic events in cancer patients (Saha and Anirvan, 2020). Q96PL1_SG3A2 is highly expressed and shows antifibrotic activity in the lungs (Cai et al., 2014).

**FIGURE 3:**
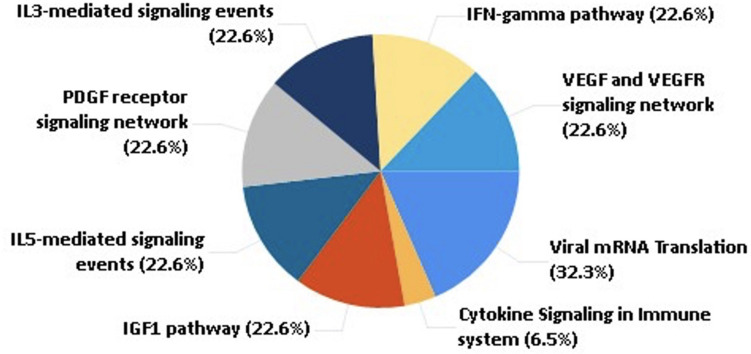
Pathway analysis of the selected 45 proteins.

These shortlisted proteins were further analyzed to understand their role in human physiology and COVID-19 prognosis. From the pathway analysis, we found that the selected 45 proteins are associated with pathways such as the IFN-gamma pathway, IL5 and IL3 mediating signaling events, cytokine, chemokine, and VEGF signaling, as shown in Figure 3.

### Identification of Potential Drug Targets Among the Shortlisted Proteins

To date, no reliable drug has been approved to treat COVID-19. From the drug target database (Supplementary Table 4), we were able to identify clinically used drugs that target 18 proteins among the shortlisted 45 proteins. The maximum number of drugs was found to target growth factor associated proteins, i.e., VGFR2 and EGFR, followed by FA7 and ANGP2 (Supplementary Figure 1). It is observed that during viral infection through respiratory viruses, EGFR gets activated via the NADPH oxidase signaling pathway in the airway epithelium. The activation of EGFR causes suppression of IFN regulatory factor (IRF) 1-dependent CXCL10 production showing their role in antiviral defense (Kalinowski et al., 2014).

### The Development and Utility of the CovidPrognosis Webserver

**FIGURE 4:**
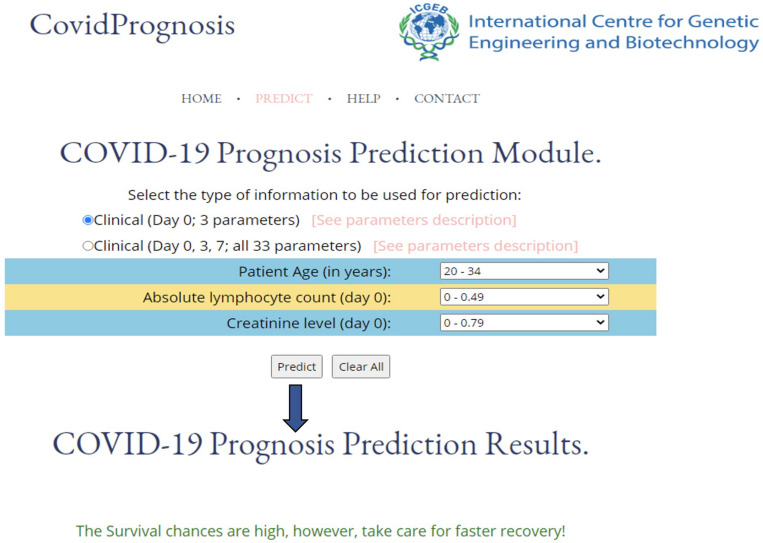
A screenshot showing the functionality of the CovidPrognosis webserver with three clinical parameters for Day 0.

The utility of a machine learning–based method relies upon its ease of use. Therefore, to enhance the real-life usage of the developed prediction models by researchers or clinicians, we have developed the webserver CovidPrognosis. The webserver is freely available for scientific use and clinical validation at http://14.139.62.220/covidprognosis/. In the current version, the users can input three parameters for Day 0 or 33 parameters for Days 0, 3, and 7. The survival chances of the patient, represented by the input parameters, are predicted based on the user-supplied values. A detailed description of the clinical parameters is available on the CovidPrognosis webserver’s website at http://14.139.62.220/covidprognosis/help.php. Day 0 denotes the day on which the patient was admitted to a hospital, while Days 3 and 7 represent the third and seventh day after hospitalization, respectively. The Day 0–based model helps in the early estimation of the seriousness of the case, while the days 0–7-based model may prove useful while monitoring the patient’s health status at the time of hospital stay. Figure 4 shows the prediction results by the CovidPrognosis webserver’s three clinical parameters-based model using Day 0 clinical information of a COVID-19 patient. The webserver may prove to be a valuable resource for researchers and clinicians for independent validation and further improvement.

## Discussion

COVID-19 is caused by the novel coronavirus SARS-CoV-2 that belongs to the SARS-CoV and MERS family of viruses. To date, the disease has led to millions of deaths worldwide. COVID-19 can be diagnosed by real-time PCR (RT-PCR), chest X-ray images, CT scan images, and serological blood tests (Augustine et al., 2020, p. 19). However, these diagnostic methods have low accuracy with a high false-positive rate of prediction (Surkova et al., 2020; To et al., 2020) and cannot help distinguish patients with different severity of illness. In addition to the respiratory illness, COVID-19 can cause many other illnesses such as kidney failure, heart disease, and venous thromboembolism and may damage the CNS leading to mortality (Kollias et al., 2020; Larsen et al., 2020; Shi et al., 2020; Wu et al., 2020).

The most common clinical abnormalities observed in COVID-19 positive patients are lymphopenia, leukopenia, thrombocytopenia, elevated CRP and inflammatory markers, elevated cardiac biomarkers, decreased albumin, and abnormal renal and liver function (Paranjpe et al., 2020; Zhu et al., 2020). The increase in SARS-CoV-2 spread and mortality has motivated researchers to develop vaccines or antiviral drugs. Similarly, clinicians too are trying different treatment strategies to improve prognosis, reduce treatment period, and alleviate the suffering of COVID-19 patients. Therefore, it is necessary to identify factors/biomarkers associated with the patients’ mortality and survival on available patient datasets to reduce the mortality rate.

Based on clinical parameters, researchers have identified several biomarkers (using an ML-based approach) like using a multivariable logistic regression model. Yao Y. et al. (2020) showed that the value of D-dimer > 2mg/L was associated with mortality among COVID-19 patients. The group has observed a significant correlation between D-dimer levels and disease severity measured by the CT, oxygenation index, and clinical staging. Another group, Yan et al. (2020a), identified lactic dehydrogenase (LDH), lymphocyte, and high-sensitivity C-reactive protein (hs-CRP) that were associated with the survival of individual patients. Similarly, in the present study, we have applied ML-based prediction on a cohort of 306 COVID positive patients with 33 clinical parameters and 1,428 protein expression values. From the number of WEKA models on clinical data, RandomSubSpace and IterativeClassifierOptimizer perform best with the accuracy of 87.37 and 84.32%, respectively. These models identified nine shortlisted features from among 33 clinical parameters, namely, age category, absolute lymphocyte count (Day 0), creatinine level (Day 0), preexisting heart disease(s), preexisting hypertension, preexisting kidney disease(s), D-dimer level (Day 0), GI symptoms, and cardiac event-troponin level 72 h (hs-cTn = > 100 within the first 72 h of presentation). Of the nine shortlisted clinical parameters, D-dimer, lymphocyte count, and kidney disease are reported to play an important role in the survival prediction of COVID-19 patients, thus validating the findings of the present study (Cheng et al., 2020; Pan et al., 2020; Yan et al., 2020a). Moreover, some previously not identified clinical parameters such as creatinine, age, and cardiac troponin, along with GI symptoms, heart disease, and hypertension, could predict the COVID-19 prognosis and disease severity.

While employing LogitBoost on 1428 protein expression data, survival prediction models were able to achieve an accuracy of 83.52% with sensitivity (%), specificity (%), MCC, and ROC values of 82.93, 84, 0.67, and 0.868, respectively. However, the accuracy was further improved after applying the feature selection algorithms (available in WEKA), and the highest accuracy of 89.01% (with the balanced dataset) was achieved with sensitivity (%), specificity (%), MCC, and ROC values of 92.68, 86, 0.78, and 0.953, respectively. Thus, the model led to identifying 45 proteins enriched in various pathways such as angiogenesis, interleukin, cytokine, chemokine, and VEGF signaling. The enrichment of host immune system pathways suggested that SARS-CoV-2 uses the host immune system defense mechanism to hijack the body’s mucous membrane cells.

Shen et al. have identified 93 proteins associated with the severity of COVID-19 disease based on the data of 46 COVID-positive patients using machine learning models (Bojkova et al., 2020; Qiu et al., 2020; Shen et al., 2020). Interestingly, some of the shortlisted 45 proteins, such as PROC, IL16, EGFR, ANGP2, APOP1, coagulation factor VII, and FEUTB (identified in the present study), are already well reported in the literature for their role in the disease prognosis and severity, thus validating the current findings (Bojkova et al., 2020; Qiu et al., 2020; Shen et al., 2020; Shu et al., 2020; Yin et al., 2020). In our analysis, other protein classes such as different growth factors and phospholipase factors are newly discovered, which can be explored further for their role in disease severity. The role of phospholipase A2 in the inhibition of coronavirus replication is well established by EM and confocal microscopy, which can also be confirmed for SARS-CoV-2 (Müller et al., 2017).

From the drug-target network construction, it is observed that FDA-approved drugs target growth factor associated proteins, i.e., VGFR2 and EGFR, followed by FA7 and ANGP2, suggesting their potential implication in drug repurposing.

From the present study, we show that the ML-based prediction/classification models can efficiently help in the prognosis of COVID-19 patients based upon identified clinical and protein biomarkers associated with COVID-19 severity/survival. The clinicians and researchers can test new COVID-19 cases to predict the patients who are likely to survive within 28 days after hospitalization. The results obtained from the ML-based techniques may also lead to the biomarker discovery for COVID-19 for early prognosis, potentially reducing mortality rate and may also serve as useful drug targets.

To increase the utility of the present work, we have developed an easy-to-use CovidPrognosis webserver to assist researchers and clinicians in quickly evaluating the machine learning model or identifying the prognostic biomarkers associated with the survival or death of COVID-19 patients. The webserver is available at http://14.139.62.220/covidprognosis/. The current version of the model is a proof of concept that machine learning–based prognostic tools can be developed. The CovidPrognosis webserver will be regularly updated with the latest COVID-19 datasets in order to increase its efficiency, reliability, and utility.

## Data Availability Statement

Publicly available datasets were analyzed in this study. This data can be found here: https://www.olink.com/mgh-covid-study/.

## Author Contributions

DG, AS, and RS conceptualized the study, analyzed the data, and prepared the manuscript. AS carried out the machine learning studies. All authors reviewed and approved the final version.

## Conflict of Interest

The authors declare that the research was conducted in the absence of any commercial or financial relationships that could be construed as a potential conflict of interest.

## Footnotes

1 https://covid19.who.int/

2 http://14.139.62.220/covidprognosis/supple.php

## Supplementary Material

Supplementary Figure 1 Drug–target network of the top proteins among the selected 45 proteins.

Supplementary Table 1 Clinical information or parameters used for the generation of clinical information-based models.

Supplementary Table 2 Description of shortlisted 45 proteins useful in the classification of survived vs. died COVID-19 patients.

Supplementary Table 3 NPX expression values for 45 shortlisted proteins.

Supplementary Table 4 Drug–target interactions retrieved from TTD.
