# Supplementary figures and images for: Machine Learning Assisted Prediction of Prognostic Biomarkers Associated With COVID-19, Using Clinical and Proteomics Data
Source: Front Genet. 2021 May 20;12:636441. doi: 10.3389/fgene.2021.636441 (PMC8175075; doi:10.3389/fgene.2021.636441)

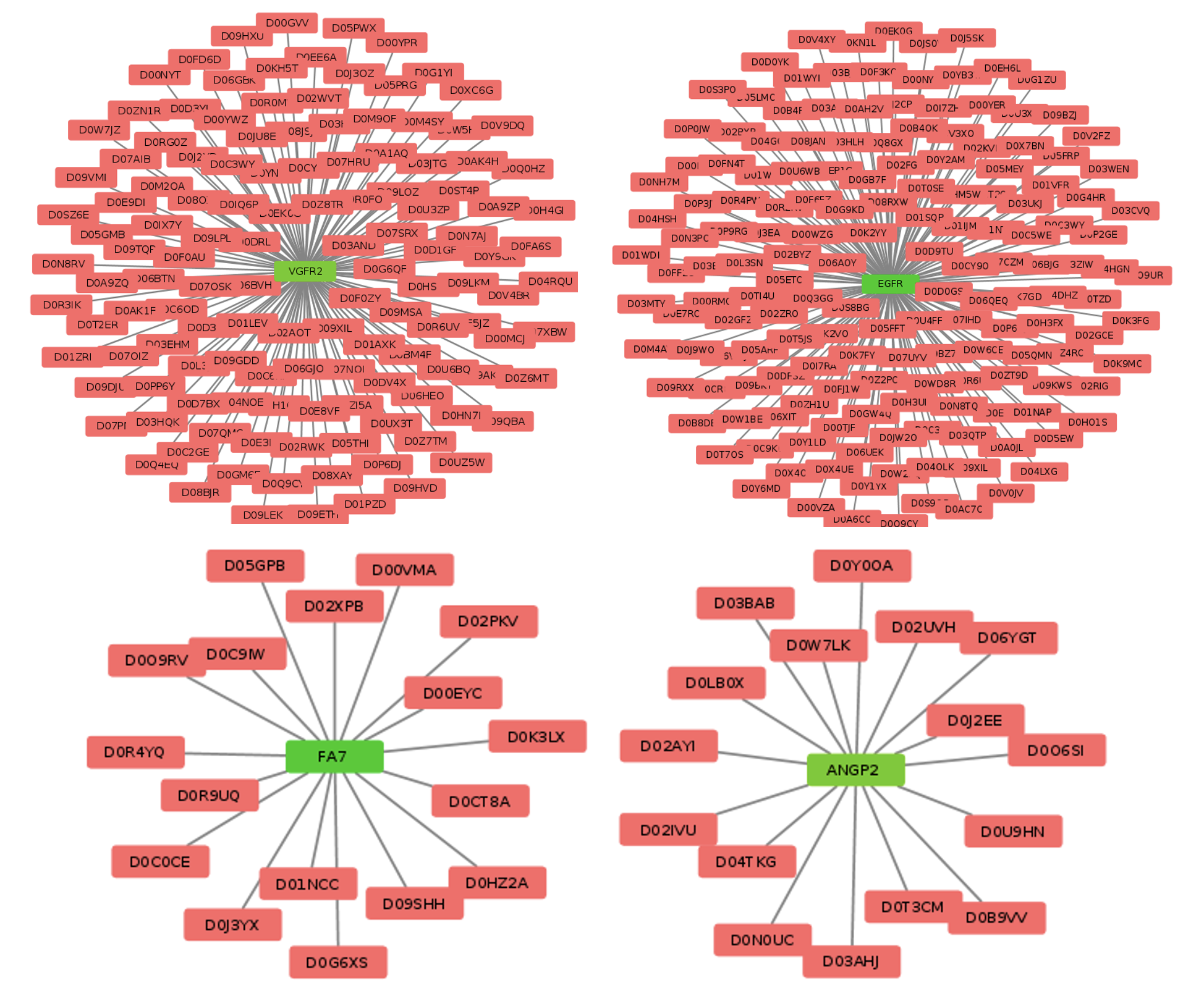

Supplement: Supplementary Figure 1 — Drug–target network of the top proteins among the selected 45 proteins. [file Image_1.PNG]
