# Supplementary material for: Machine Learning Assisted Prediction of Prognostic Biomarkers Associated With COVID-19, Using Clinical and Proteomics Data
Source: Front Genet. 2021 May 20;12:636441. doi: 10.3389/fgene.2021.636441 (PMC8175075; doi:10.3389/fgene.2021.636441)
Supplement: Supplementary Table 1 — Clinical information or parameters used for the generation of clinical information-based models. [file Table_1.DOCX]

**Table S1.** Clinical information or parameters used for generation of clinical information-based models.

| **Sr. No.** | **Clinical parameter** | **Description and numeric codes assigned to categorize values** |
| --- | --- | --- |
| 1 | subject_id | Subject ID |
| 2 | COVID | COVID status (tested positive prior to enrollment or during hospitalization) 0 = negative 1= positive |
| 3 | Age cat | Age category 1 = 20-34 2 = 36-49 3 = 50-64 4 = 65-79 5 = 80+ |
| 4 | BMI cat | Body mass index: 0 = <18.5 (underweight) 1 = 18.5-24.9 (normal) 2 = 25.0-29.9 (overweight) 3 = 30.0-39.9 (obese) 4 = >=40 (severely obese) 5 = Unknown |
| 5 | HEART | Pre-existing heart disease – HEART - (coronary artery disease, congestive heart failure, valvular disease) 0 = No 1 = Yes |
| 6 | LUNG | Pre-existing lung disease – LUNG - (asthma, COPD, requiring home O2, any chronic lung condition) 0 = No 1 = Yes |
| 7 | KIDNEY | Pre-existing kidney disease – KIDNEY - (chronic kidney disease, baseline creatinine >1.5, ESRD) 0 = No 1 = Yes |
| 8 | DIABETES | Pre-existing diabetes – DIABETES - (pre-diabetes, insulin and non-insulin dependent diabetes) 0 = No 1 = Yes |
| 9 | HTN | Pre-existing hypertension - HTN 0 = No 1 = Yes |
| 10 | IMMUNO | Pre-existing immunocompromised condition – IMMUNO (active cancer, chemotherapy, transplant, immunosuppressant agents, aspenic) 0 = No 1 = Yes |
| 11 | Resp_Symp | Respiratory symptoms – Symp_Resp (sore throat, congestion, productive or dry cough, shortness of breath or hypoxia, or chest pain) 0 = No 1 = Yes |
| 12 | Fever_Sympt | Febrile symptom (Yes = 1 No = 0) |
| 13 | GI_Symp | Any GI related symptoms at presentation (abdominal pain, nausea, vomiting, diarrhea) (Yes = 1 No = 0) |
| 14 | Trop_72h | Cardiac event – Trop_72h - (hs-cTn =>100 within first 72 hours of presentation) 0 = No 1 = Yes |
| **Other Clinical Parameters (Day-wise)** | | |
| **Day 0** | | |
| 15 | abs_neut_0_cat | Absolute neutrophil count day 0 category: 1 = 0-0.99 2 = 1.0-3.99 3 = 4.0-7.99 4 = 8.0-11.99 5 = 12+ |
| 16 | abs_lymph_0_cat | Absolute lymphocyte count day 0 category: 1 = 0-0.49 2 = 0.50-0.99 3 = 1.00-1.49 4 = 1.50-1.99 5 = 2+ |
| 17 | abs_mono_0_cat | Absolute monocyte day 0 category 1 = 0-0.24 2 = 0.25-0.49 3 = 0.50-0.74 4 = 0.75-0.99 5 = 1.0+ |
| 18 | creat_0_cat | Creatinine day 0 category 1 = 0-0.79 2 = 0.80-1.19 3 = 1.20-1.79 4 = 1.80-2.99 5 = 3+ |
| 19 | crp_0_cat | c-reactive protein day 0 category: 1 = 0-19.9 2 = 20-59.0 3 = 60-99.9 4 = 100-179 5 = 180+ |
| 20 | ddimer_0_cat | D-dimer day 0 category: 1 = 0-499 2 = 500-999 3 = 1000-1999 4 = 2000-3999 5 = 4000+ |
| 21 | ldh_0_cat | Lactate dehydrogenase day 0 category: 1 = 0-200 2 = 200-299 3 = 300-399 4 = 400-499 5 = 500+ |
| **Day 3** | | |
| 22 | abs_neut_3_cat | Absolute neutrophil count day 3 category: 1 = 0-0.99 2 = 1.0-3.99 3 = 4.0-7.99 4 = 8.0-11.99 5 = 12+ |
| 23 | abs_lymph_3_cat | Absolute lymphocyte count day 3 category: 1 = 0-0.49 2 = 0.50-0.99 3 = 1.00-1.49 4 = 1.50-1.99 5 = 2+ |
| 24 | abs_mono_3_cat | Absolute monocyte count day 3 category: 1 = 0-0.24 2 = 0.25-0.49 3 = 0.50-0.74 4 = 0.75-0.99 5 = 1.0+ |
| 25 | creat_3_cat | Creatinine day 3 category 1 = 0-0.79 2 = 0.80-1.19 3 = 1.20-1.79 4 = 1.80-2.99 5 = 3+ |
| 26 | crp_3_cat | c-reactive protein day 3 category: 1 = 0-19.9 2 = 20-59.0 3 = 60-99.9 4 = 100-179 5 = 180+ |
| 27 | ddimer_3_cat | D-dimer day 3 category: 1 = 0-499 2 = 500-999 3 = 1000-1999 4 = 2000-3999 5 = 4000+ |
| 28 | ldh_3_cat | Lactate dehydrogenase day 3 category: 1 = 0-200 2 = 200-299 3 = 300-399 4 = 400-499 5 = 500+ |
| **Day 7** | | |
| 29 | abs_neut_7_cat | Absolute neutrophil count day 7 category: 1 = 0-0.99 2 = 1.0-3.99 3 = 4.0-7.99 4 = 8.0-11.99 5 = 12+ |
| 30 | abs_lymph_7_cat | Absolute lymphocyte count day 7 category: 1 = 0-0.49 2 = 0.50-0.99 3 = 1.00-1.49 4 = 1.50-1.99 5 = 2+ |
| 31 | abs_mono_7_cat | Absolute monocyte count day 7 category: 1 = 0-0.24 2 = 0.25-0.49 3 = 0.50-0.74 4 = 0.75-0.99 5 = 1.0+ |
| 32 | creat_7_cat | Creatinine day 7 category 1 = 0-0.79 2 = 0.80-1.19 3 = 1.20-1.79 4 = 1.80-2.99 5 = 3+ |
| 33 | crp_7_cat | c-reactive protein day 7 category: 1 = 0-19.9 2 = 20-59.0 3 = 60-99.9 4 = 100-179 5 = 180+ |
| 34 | ddimer_7_cat | D-dimer day 3 category: 1 = 0-499 2 = 500-999 3 = 1000-1999 4 = 2000-3999 5 = 4000+ |
| 35 | ldh_7_cat | Lactate dehydrogenase day 7 category: 1 = 0-200 2 = 200-299 3 = 300-399 4 = 400-499 5 = 500+ |
