# Supplementary material for: Machine Learning Assisted Prediction of Prognostic Biomarkers Associated With COVID-19, Using Clinical and Proteomics Data
Source: Front Genet. 2021 May 20;12:636441. doi: 10.3389/fgene.2021.636441 (PMC8175075; doi:10.3389/fgene.2021.636441)
Supplement: Supplementary Table 2 — Description of shortlisted 45 proteins useful in the classification of survived vs. died COVID-19 patients. [file Table_2.DOCX]

**Table S2.** Description of shortlisted 45 proteins useful in the classification of survived vs. died COVID-19 patients.

| **UniProt ID** | **Gene Name** | **Description** |
| --- | --- | --- |
| Q9Y653 | AGRG1 | Adhesion G-protein coupled receptor G1 (G-protein coupled receptor 56) (Protein TM7XN1) [Cleaved into: ADGRG1 N-terminal fragment (ADGRG1 NT) (GPR56 N-terminal fragment) (GPR56 NT) (GPR56(N)) (GPR56 extracellular subunit) (GPR56 subunit alpha); ADGRG1 C-terminal fragment (ADGRG1 CT) (GPR56 C-terminal fragment) (GPR56 CT) (GPR56(C)) (GPR56 seven-transmembrane subunit) (GPR56 7TM) (GPR56 subunit beta)] |
| O95841 | ANGL1 | Angiopoietin-related protein 1 (Angiopoietin-3) (ANG-3) (Angiopoietin-like protein 1) |
| O15123 | ANGP2 | Angiopoietin-2 (ANG-2) |
| O95445 | APOM | Apolipoprotein M (Apo-M) (ApoM) (Protein G3a) |
| Q76LX8 | ATS13 | A disintegrin and metalloproteinase with thrombospondin motifs 13 (ADAM-TS 13) (ADAM-TS13) (ADAMTS-13) (EC 3.4.24.87) (von Willebrand factor-cleaving protease) (vWF-CP) (vWF-cleaving protease) |
| P55291 | CAD15 | Cadherin-15 (Cadherin-14) (Muscle cadherin) (M-cadherin) |
| P22676 | CALB2 | Calretinin (CR) (29 kDa calbindin) |
| P13500 | CCL2 | C-C motif chemokine 2 (HC11) (Monocyte chemoattractant protein 1) (Monocyte chemotactic and activating factor) (MCAF) (Monocyte chemotactic protein 1) (MCP-1) (Monocyte secretory protein JE) (Small-inducible cytokine A2) |
| O76076 | CCN5 | CCN family member 5 (Connective tissue growth factor-like protein) (CTGF-L) (Connective tissue growth factor-related protein 58) (WNT1-inducible-signaling pathway protein 2) (WISP-2) |
| P29017 | CD1C | T-cell surface glycoprotein CD1c (CD antigen CD1c) |
| P36222 | CH3L1 | Chitinase-3-like protein 1 (39 kDa synovial protein) (Cartilage glycoprotein 39) (CGP-39) (GP-39) (hCGP-39) (YKL-40) |
| Q13231 | CHIT1 | Chitotriosidase-1 (EC 3.2.1.14) (Chitinase-1) |
| P09496 | CLCA | Clathrin light chain A (Lca) |
| P02462 | CO4A1 | Collagen alpha-1(IV) chain [Cleaved into: Arresten] |
| P13611 | CSPG2 | Versican core protein (Chondroitin sulfate proteoglycan core protein 2) (Chondroitin sulfate proteoglycan 2) (Glial hyaluronate-binding protein) (GHAP) (Large fibroblast proteoglycan) (PG-M) |
| P00533 | EGFR | Epidermal growth factor receptor (EC 2.7.10.1) (Proto-oncogene c-ErbB-1) (Receptor tyrosine-protein kinase erbB-1) |
| P08709 | FA7 | Coagulation factor VII (EC 3.4.21.21) (Proconvertin) (Serum prothrombin conversion accelerator) (SPCA) (Eptacog alfa) [Cleaved into: Factor VII light chain; Factor VII heavy chain] |
| Q9UGM5 | FETUB | Fetuin-B (16G2) (Fetuin-like protein IRL685) (Gugu) |
| P12034 | FGF5 | Fibroblast growth factor 5 (FGF-5) (Heparin-binding growth factor 5) (HBGF-5) (Smag-82) |
| O14793 | GDF8 | Growth/differentiation factor 8 (GDF-8) (Myostatin) |
| Q92820 | GGH | Gamma-glutamyl hydrolase (EC 3.4.19.9) (Conjugase) (GH) (Gamma-Glu-X carboxypeptidase) |
| P55259 | GP2 | Pancreatic secretory granule membrane major glycoprotein GP2 (Pancreatic zymogen granule membrane protein GP-2) (ZAP75) |
| Q8IU54 | IFNL1 | Interferon lambda-1 (IFN-lambda-1) (Cytokine Zcyto21) (Interleukin-29) (IL-29) |
| P01591 | IGJ | Immunoglobulin J chain (Joining chain of multimeric IgA and IgM) |
| Q9P0M4 | IL17C | Interleukin-17C (IL-17C) (Cytokine CX2) |
| P10145 | IL8 | Interleukin-8 (IL-8) (C-X-C motif chemokine 8) (Chemokine (C-X-C motif) ligand 8) (Emoctakin) (Granulocyte chemotactic protein 1) (GCP-1) (Monocyte-derived neutrophil chemotactic factor) (MDNCF) (Monocyte-derived neutrophil-activating peptide) (MONAP) (Neutrophil-activating protein 1) (NAP-1) (Protein 3-10C) (T-cell chemotactic factor) [Cleaved into: MDNCF-a (GCP/IL-8 protein IV) (IL8/NAP1 form I); Interleukin-8 ((Ala-IL-8)77) (GCP/IL-8 protein II) (IL-8(1-77)) (IL8/NAP1 form II) (MDNCF-b); IL-8(5-77); IL-8(6-77) ((Ser-IL-8)72) (GCP/IL-8 protein I) (IL8/NAP1 form III) (Lymphocyte-derived neutrophil-activating factor) (LYNAP) (MDNCF-c) (Neutrophil-activating factor) (NAF); IL-8(7-77) (GCP/IL-8 protein V) (IL8/NAP1 form IV); IL-8(8-77) (GCP/IL-8 protein VI) (IL8/NAP1 form V); IL-8(9-77) (GCP/IL-8 protein III) (IL8/NAP1 form VI)] |
| Q14767 | LTBP2 | Latent-transforming growth factor beta-binding protein 2 (LTBP-2) |
| Q8TD46 | MO2R1 | Cell surface glycoprotein CD200 receptor 1 (CD200 cell surface glycoprotein receptor) (Cell surface glycoprotein OX2 receptor 1) |
| P14555 | PA2GA | Phospholipase A2, membrane associated (EC 3.1.1.4) (GIIC sPLA2) (Group IIA phospholipase A2) (Non-pancreatic secretory phospholipase A2) (NPS-PLA2) (Phosphatidylcholine 2-acylhydrolase 2A) |
| P01127 | PDGFB | Platelet-derived growth factor subunit B (PDGF subunit B) (PDGF-2) (Platelet-derived growth factor B chain) (Platelet-derived growth factor beta polypeptide) (Proto-oncogene c-Sis) (Becaplermin) |
| P04070 | PROC | Vitamin K-dependent protein C (EC 3.4.21.69) (Anticoagulant protein C) (Autoprothrombin IIA) (Blood coagulation factor XIV) [Cleaved into: Vitamin K-dependent protein C light chain; Vitamin K-dependent protein C heavy chain; Activation peptide] |
| P58294 | PROK1 | Prokineticin-1 (Endocrine-gland-derived vascular endothelial growth factor) (EG-VEGF) (Mambakine) |
| P26022 | PTX3 | Pentraxin-related protein PTX3 (Pentaxin-related protein PTX3) (Tumor necrosis factor alpha-induced protein 5) (TNF alpha-induced protein 5) (Tumor necrosis factor-inducible gene 14 protein) (TSG-14) |
| Q16769 | QPCT | Glutaminyl-peptide cyclotransferase (EC 2.3.2.5) (Glutaminyl cyclase) (QC) (sQC) (Glutaminyl-tRNA cyclotransferase) (Glutamyl cyclase) (EC) |
| P50749 | RASF2 | Ras association domain-containing protein 2 |
| Q96PL1 | SG3A2 | Secretoglobin family 3A member 2 (Pneumo secretory protein 1) (PnSP-1) (Uteroglobin-related protein 1) |
| Q9Y3P8 | SIT1 | Signaling threshold-regulating transmembrane adapter 1 (SHP2-interacting transmembrane adapter protein) (Suppression-inducing transmembrane adapter 1) (gp30/40) |
| Q14515 | SPRL1 | SPARC-like protein 1 (High endothelial venule protein) (Hevin) (MAST 9) |
| P13385 | TDGF1 | Teratocarcinoma-derived growth factor 1 (Cripto-1 growth factor) (CRGF) (Epidermal growth factor-like cripto protein CR1) |
| P01135 | TGFA | Protransforming growth factor alpha [Cleaved into: Transforming growth factor alpha (TGF-alpha) (EGF-like TGF) (ETGF) (TGF type 1)] |
| Q15633 | TRBP2 | RISC-loading complex subunit TARBP2 (TAR RNA-binding protein 2) (Trans-activation-responsive RNA-binding protein) |
| O60635 | TSN1 | Tetraspanin-1 (Tspan-1) (Tetraspan NET-1) (Tetraspanin TM4-C) |
| Q8NBZ7 | UXS1 | UDP-glucuronic acid decarboxylase 1 (EC 4.1.1.35) (UDP-glucuronate decarboxylase 1) (UGD) (UXS-1) |
| P35968 | VGFR2 | Vascular endothelial growth factor receptor 2 (VEGFR-2) (EC 2.7.10.1) (Fetal liver kinase 1) (FLK-1) (Kinase insert domain receptor) (KDR) (Protein-tyrosine kinase receptor flk-1) (CD antigen CD309) |
| Q96NZ8 | WFKN1 | WAP, Kazal, immunoglobulin, Kunitz and NTR domain-containing protein 1 (Growth and differentiation factor-associated serum protein 2) (GASP-2) (hGASP-2) (WAP, follistatin, immunoglobulin, Kunitz and NTR domain-containing protein) |
